# Pain and Posttraumatic Stress Symptom Clusters: A Cross-Lagged Study

**DOI:** 10.3389/fpsyg.2021.669231

**Published:** 2021-05-31

**Authors:** Vivian de Vries, Alette E. E. de Jong, Helma W. C. Hofland, Nancy E. Van Loey

**Affiliations:** ^1^Department of Medical Psychology, Red Cross Hospital, Beverwijk, Netherlands; ^2^Burn Center, Red Cross Hospital, Beverwijk, Netherlands; ^3^Association of Dutch Burn Centers, Department Psychological and Nursing Research, Beverwijk, Netherlands; ^4^Burn Center, Maasstad Hospital, Rotterdam, Netherlands; ^5^Association of Dutch Burn Centers, Department Psychological and Nursing Research, Rotterdam, Netherlands; ^6^Department of Clinical Psychology, Utrecht University, Utrecht, Netherlands

**Keywords:** burns, pain, cross-lagged analyses, posttraumatic stress disorder, mutual maintenance

## Abstract

Pain and posttraumatic stress disorder (PTSD) frequently co-occur but underlying mechanisms are not clear. This study aimed to test the development and maintenance of pain and PTSD symptom clusters, i.e., intrusions, avoidance, and hyperarousal. The longitudinal study included 216 adults with burns. PTSD symptom clusters, indexed by the Impact of Event Scale-Revised (IES-R), and pain, using a graphic numerical rating scale (GNRS), were measured during hospitalization, 3 and 6 months post-burn. Cross-lagged panel analysis was used to test the relationships between pain and PTSD symptom clusters. Cross-lagged results showed that in-hospital intrusions predicted pain and avoidance 3 months post-burn. In-hospital pain predicted intrusions and avoidance 3 months post-burn and a trend was found for hyperarousal (90% CI). In the second wave, intrusions predicted pain and hyperarousal. Pain predicted hyperarousal. This study provides support for an entangled relationship between pain and PTSD symptoms, and particularly subscribes the role of intrusions in this bidirectional relationship. To a lesser extent, hyperarousal was unidirectionally related to pain. These results may subscribe the driving role of PTSD, particularly intrusions, which partly supports the Perpetual Avoidance Model.

## Introduction

A burn injury is associated with substantial pain and patients with burns are at risk of developing posttraumatic stress disorder (PTSD) as a result of being exposed to a traumatic event (Richardson and Mustard, 2009; Hobbs, 2015). A substantial proportion of burn survivors, estimated at 52%, reported pain on average 12 years after the injury (Dauber et al., 2002). PTSD is prevalent in ~10% of patients who were admitted to a burn center and an additional 15% of burn survivors experience subclinical PTSD symptoms 1–4 years later (Dyster-Aas et al., 2008; Van Loey et al., 2008). Both pain and PTSD affect functioning up to 2 years post-burn (Corry et al., 2010). A better understanding of how these concepts influence each other may inform clinical practice as to how to prevent these long-term adverse consequences.

The high co-morbidity between (chronic) pain and PTSD is well-documented (Siqveland et al., 2017; Ravn et al., 2018). While the exact nature of the relationship between pain and PTSD symptoms is still largely unknown, there are several theories on how the concepts might be associated. Sharp and Harvey (2001) proposed the Mutual Maintenance Model of PTSD and pain. This model incorporates several dysfunctional cognitive, behavioral and affective mechanisms that may exacerbate both PTSD and pain. Another theory, the Perpetual Avoidance Model (Liedl and Knaevelsrud, 2008), proposes that PTSD is driving the presence of pain, not vice versa. The Perpetual Avoidance Model assumes that intrusions following a traumatic event induce a vicious circle of hyperarousal, pain, catastrophizing, avoidance and more intrusions. The model also assumes that hyperarousal induced by intrusions directly leads to avoidance which, in turn, triggers intrusions. A central role for avoidance behavior in pain maintenance is also proposed in the Fear-Avoidance Model (Vlaeyen and Linton, 2012), one of the most influential models to explain chronic pain. The model describes how the dysfunctional interpretation of pain as threatening leads to fear and avoidance behavior that in turn increases pain. Although this model does not specifically addresses PTSD, it is well-established that avoidance behavior plays a central role in both pain and PTSD.

Support for the mutual maintenance between pain and PTSD symptom clusters was found in a sample of trauma patients (Liedl et al., 2010). In this study, the relationship between acute pain and 12-month pain was mediated by hyperarousal symptoms at 3 months. The relationship between baseline hyperarousal and intrusion symptoms, and later 12-month hyperarousal and intrusion symptoms, was mediated by 3-month pain levels. In a sample of injured accident survivors (Jenewein et al., 2009), support for the Mutual Maintenance Model was found in the early aftermath of the accident. Six to 12 months post-accident the findings demonstrated that higher PTSD symptom levels were associated with increased pain intensity, but not vice versa. In a study in children with traumatic brain injury (Brown et al., 2014), both the Mutual Maintenance Model and the Perpetual Avoidance Model fitted the data well; it was concluded that PTSD drives the presence of pain. However, a systematic review of cross-lagged studies did not find unequivocal support for the theoretical framework of mutual maintenance (Ravn et al., 2018). Both bidirectional and unidirectional associations between PTSD symptomatology and pain were reported over time, with a central role for hyperarousal and intrusions. Taken together, empirical results vary widely and only partly support theoretical assumptions. The diverse results may relate to e.g., differences in trauma types, the use of total PTSD vs. PTSD symptom cluster and, symptom levels of pain and PTSD; particularly samples with high symptom levels may support mutual maintenance, as put forward by Ravn et al. (2018). Therefore, more studies are required to discern the direction of the relationship in different populations. Moreover, studies that investigate separate PTSD symptom clusters may provide insight in the role of specific symptoms, as proposed in the Perpetual Avoidance Model and Fear-Avoidance Model.

The present study aimed to test the cross-lagged pathways of PTSD symptom clusters and pain in a population of burn survivors starting in-hospital with a follow-up of 6 months. A saturated cross-lagged panel model was tested, i.e., the starting point was to test mutual maintenance between pain and PTSD symptom clusters. This approach is in line with Liedl et al. (2010). This approach was deemed most suitable because, in empirical studies, there is no univocal evidence on the relationship between pain and separate PTSD symptom clusters. Additionally, to our knowledge, these relationships were not examined in burn populations.

## Method

### Participants

Study participants were adult patients with burns admitted to a burn center in the Netherlands or Belgium between 2009 and 2011. Patients were eligible for participation if they were 18 years or older, had proficiency in Dutch and stayed in hospital >24 h. Patients were excluded from participation if they suffered from cognitive impairment, experienced psychosis or when the burns were the result of self-harm or a suicide attempt. The results are part of a larger prospective study on pain, PTSD symptoms and quality of life (Bosmans et al., 2015). The study variables, be it the total PTSD score and pain measured in-hospital and at 3 months post-burn, were previously reported (Van Loey et al., 2018).

A total of 303 patients met the inclusion criteria, of which 87 could not be included. This was either due to refusal to participate in the study (*N* = 58) or patients could not be included according to the study schedule because the researcher was not available or because patients were medically unstable (*N* = 29). The excluded patients did not differ from participants in terms of gender [χ^2^(1, *N* = 289) = 0.187, *p* = 0.67] and age [t_(283)_ = −0.313, *p* = 0.75] but they had a higher Total Body Surface Area (TBSA) burned [t_(299)_ = 3.140, *p* = 0.002; *M* = 13 vs. *M* = 9].

The total sample consisted of 216 patients, of which seven were excluded because of missing values on all variables. The remaining 209 (97%) were included in the model. Pain scores were provided by 123 patients (57%) at 3 months post-burn (T2) and by 119 patients (55%) at 6 months post-burn (T3). Scores on at least one of the three PTSD symptom clusters were provided by 167 (77%) and 157 patients (73%) at 3 and 6 months post-burn, respectively. To illustrate, at T2, 167 participants completed all intrusion items, but only 165 completed all avoidance and hyperarousal items. The 67 patients lost to follow-up were significantly younger (*M* = 34.99, SD = 13.46 v. *M* = 43.31 SD = 15.57) [t_(214)_ = −3.784, *p* = 0.03] and had a lower TBSA burned (*M* = 7.17 v. *M* = 9.91) [t_(214)_ = −2.213, *p* = 0.01]. The sample included mostly men (*n* = 145, 67%). The mean age was 40.7 (SD = 15.4). The mean length of stay in hospital was 17.8 days (SD = 13.9). The average TBSA burned was 9.1 (SD = 8.5) with a minimum of 1% and a maximum of 75% TBSA burned. The most frequent causes of the burns were fire and scalds (hot fluid burns).

### Measures

#### Traumatic Stress Symptoms

The Impact of Event Scale-Revised (IES-R, Weiss and Marmar, 2001) was used to assess posttraumatic stress symptoms. This scale is a self-report instrument that is frequently used to measure intrusive, avoidant and hyper arousal symptoms that are associated with a traumatic event. Patients were asked to bear in mind the burn event when completing the scale. The scale used in this study includes the 15 items of the IES (Horowitz et al., 1979) and the seven hyper arousal items of the IES-R. The scoring system of the original IES (a 4-point scale of 0-1-3-5), inquiring about the frequency of the symptoms, was maintained. This version was called the IESplus (Bosmans et al., 2015). The construct validity and reliability of the Dutch version of the IES-R showed to be acceptable across different traumatic experiences (Olde et al., 2006). Cronbach's alpha for the IESplus symptom clusters intrusions, avoidance and hyperarousal in the present sample was high at all waves (T1:0.91, 0.85, 0.81; T2:0.93, 0.88, 0.89; T3:0.92, 0.90, 0.86). Scores on the IESplus can range from 0 to 110. Both the IES and the IES-R have been validated in a burn population and have been found highly related to the diagnosis posttraumatic stress disorder (Sveen et al., 2010).

#### Pain Intensity

Pain during hospitalization was measured with an 11-point Graphic Numerical Rating Scale (GNRS) ranging from 0 (no pain) to 10 (pain as bad as you can imagine). The GNRS is part of the Brief Pain Inventory (BPI). This measure is brief, simple and easy to use for the assessment of several types of pain in both clinical and research settings (de Jong et al., 2015). The GNRS is frequently used and considered reliable, also for pain measurement following burns. For this study, we used the average of the available morning background pain ratings in the first 2 weeks post-burn as a measure of “acute burn pain.” Pain after 3 and 6 months was measured using the BPI item “average pain” scored on an 11-point GNRS enquiring about pain in the previous 24 h.

#### Demographic Information and Injury Severity

Information on age, gender, length of hospitalization, and TBSA burned was recorded from patient's medical file. TBSA burned is the estimated percentage of the body covered with partial and full thickness burns.

### Procedure

Patients were invited to participate in this longitudinal study by a local researcher during their stay in the burn center. Oral and written information about the study was provided. After the patient gave written informed consent the first questionnaires were completed during hospitalization. The follow-up was conducted by mail by a local researcher. The study was approved by institutional review boards in the Netherlands and Belgium (NL27996.094.09, B670201112923). The study was conducted in accordance with the Helsinki declaration.

### Data Analyses

To test whether there were differences between patients who did and did not complete the study, *t*-tests were used for continuous data and χ^2^ tests for dichotomous data. Pearson correlations between the variables of interest are presented to illustrate univariate relationships. These analyses and other descriptive variables were conducted with SPSS version 24 (Statistics for Windows, Version 24.0. Armonk, NY: IBM Corp).

**Figure 1:**
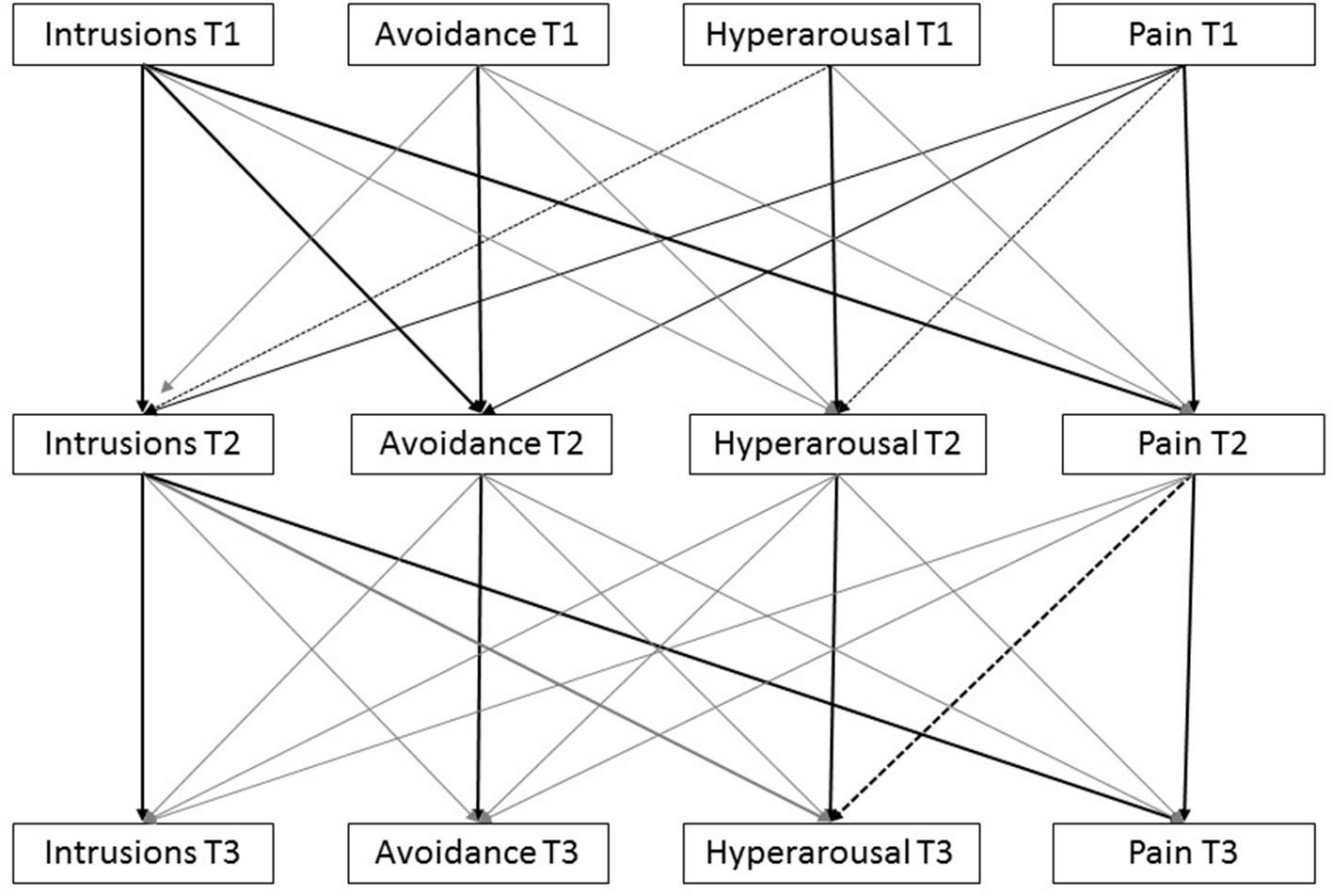
Complete model with all possible pathways connecting pain and PTSD-symptom clusters. Note that black lines are statistically significant associations, dashed lines represent trends, gray lines are not statistically significant.

The cross-lagged panel model analyses were conducted with Mplus 8.5. First, as depicted in Figure 1, the model tested the effects of the three (acute) PTSD symptom clusters and pain on the subsequent PTSD symptom clusters and pain, while controlling for the cross-sectional correlations and the stability paths, i.e., the correlations of the repeated measurements over time. Second, non-significant paths (*p* > 0.40) were constrained to zero which resulted in a model with adequate fit indices.

The model fit was evaluated using the Comparative Fit Index (CFI; Bentler and Bonett, 1980), the Tucker-Lewis Index (TLI; Tucker and Lewis, 1973), which is also known as the Non-Normed Fit Index (NNFI; Bentler and Bonett, 1980) and the Root Mean Square Error of Approximation (RMSEA). Models with TLI and CFI between 0.90 and 0.95 are deemed acceptable fit. RMSEA <0.08 is considered adequate (Kline, 2011). As values of skewness and kurtosis indicated deviations from normality in some variables, bootstrapping (*n* = 1000) was used to assess the effects. This process establishes an empirical approximation of the sampling distribution. Path estimates were considered significant in case zero was not contained in the 95% Confidence Interval (CI) and considered a trend if zero was not contained in the 90% CI. Full Information Maximum Likelihood (FIML) was applied to deal with missing data, hereby using all available data.

## Results

### Sample Characteristics and Correlations

Table 1 displays descriptive information on pain and the three symptom clusters of (acute) PTSD (intrusions, avoidance and hyperarousal) in the acute phase, after 3 and 6 months. Over time, pain intensity and PTSD symptom clusters' frequency decreased. Table 1 also presents Pearson correlations among pain and all PTSD symptom clusters at the three time points. At every time point, pain correlated significantly with the PTSD symptom clusters. The correlations between PTSD symptoms and pain became stronger over time. As expected, at the three time points the three symptom clusters of PTSD were highly correlated as well. Mean total PTSD scores were 25.5 (SD = 23.2), 21.5 (SD = 23.9), 20.6 (SD = 23.2) at the three time points, showing there was some decrease over time. As scores can range from 0 to 110, the scores are relatively low an average.

**Table 1:** Pearson's correlation coefficients and descriptive statistics.

		**1**.	**2**.	**3**.	**4**.	**5**.	**6**.	**7**.	**8**.	**9**.	**10**.	**11**.	**12**.
1	Pain T1	–											
2	Intrusions T1	0.18^*^	–										
3	Avoidance T1	0.24^**^	0.77^**^	–									
4	Hyper-arousal T1	0.26^**^	0.78^**^	0.73^**^	–								
5	Pain T2	0.54^**^	0.31^**^	0.21^*^	0.26^**^	–							
6	Intrusions T2	0.30^**^	0.61^**^	0.52^**^	0.59^**^	0.37^**^	–						
7	Avoidance T2	0.30^**^	0.57^**^	0.59^**^	0.56^**^	0.39^**^	0.83^**^	–					
8	Hyper-arousal T2	0.27^**^	0.56^**^	0.55^**^	0.62^**^	0.38^**^	0.86^**^	0.78^**^	–				
9	Pain T3	0.27^**^	0.22^*^	0.17	0.16	0.62^**^	0.45^**^	0.36^**^	0.34^**^	–			
10	Intrusions T3	0.17^*^	0.55^**^	0.47^**^	0.45^**^	0.34^**^	0.67^**^	0.57^**^	0.59^**^	0.44^**^	–		
11	Avoidance T3	0.25^**^	0.47^**^	0.57^**^	0.42^**^	0.37^**^	0.56^**^	0.67^**^	0.53^**^	0.42^**^	0.81^**^	–	
12	Hyper-arousal T3	0.23^**^	0.57^**^	0.53^**^	0.54^**^	0.46^**^	0.66^**^	0.63^**^	0.68^**^	0.47^**^	0.86^**^	0.81^**^	–
Mean		2.71	10.2	8.52	6.89	1.32	7.90	7.38	6.15	1.11	7.32	7.52	5.90
SD		1.77	9.70	8.72	6.78	1.60	9.22	8.66	7.42	1.51	8.59	9.22	6.79
Range		0–8	0–38	0–38	0–26	0–7	0–40	0–38	0–30	0–7	0–40	0–40	0–30

*T1 = in-hospital, T2 = 3 months post-burn, T3 = 6 months post-burn*.

* *p < 0.05*,

** *p < 0.01*.

### Cross-Lagged Panel Analysis

Figure 1 presents the model that was tested. CFI and TLI were within acceptable ranges, CFI = 0.975 and TLI = 0.907, but RMSEA was not (RMSEA = 0.096) and χ^2^ was statistically significant (χ^2^ = 46.77, df = 16, *p* < 0.001) which indicates some level of model misspecification. To improve the model's parsimony and to reduce the model misfit, we conservatively eliminated pathway with *p*-values >0.40 (See Appendix 1 for model estimates and *p*-values).

**Figure 2:**
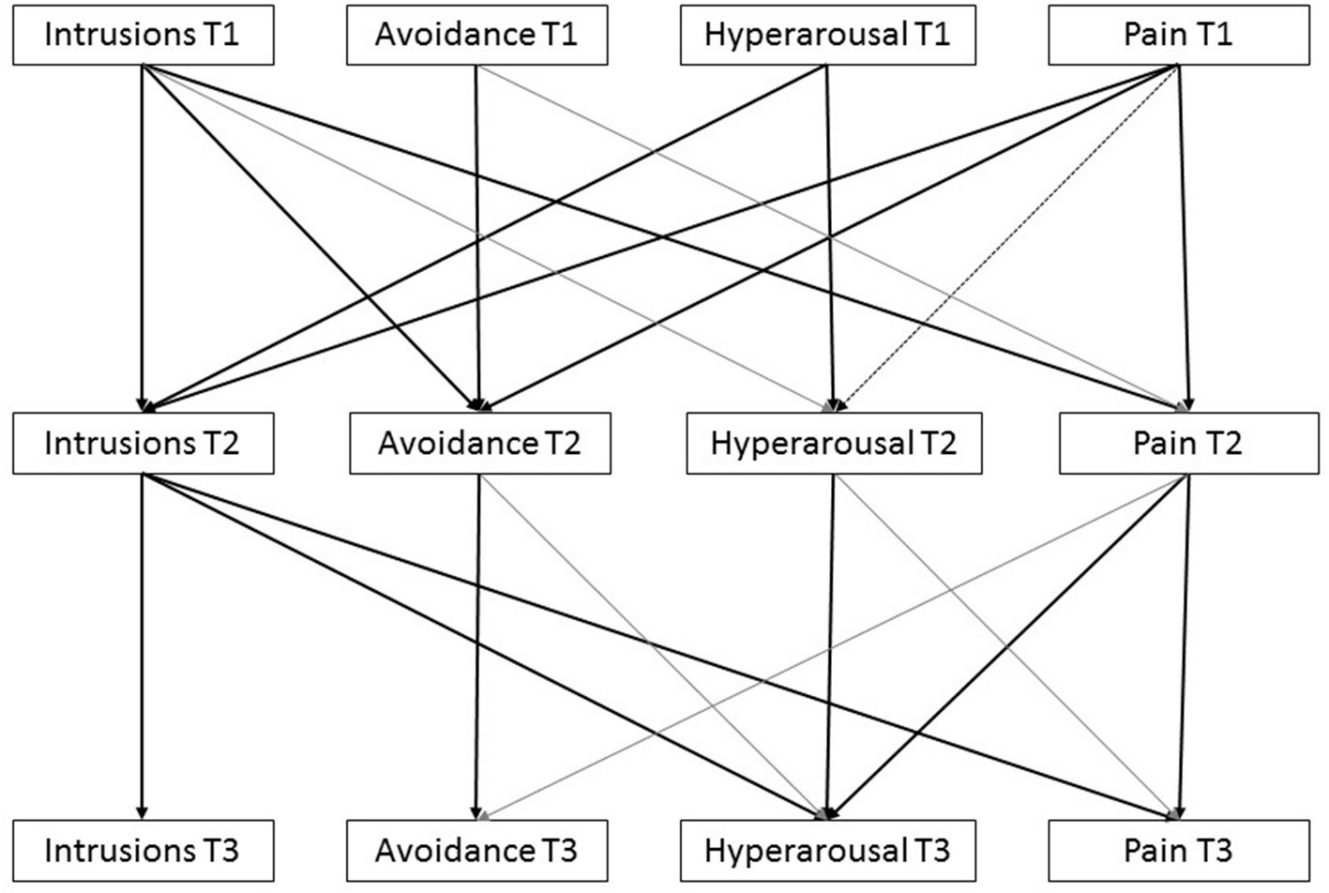
Constrained model presenting estimated pathways connecting pain and PTSD-symptom clusters. Note that black lines are statistically significant associations, gray lines are not statistically significant.

Figure 2 presents the constrained model including all tested paths between pain and the PTSD clusters. Cross-sectional pathways are also tested but hidden from view. It shows that intrusions at T1 predicted avoidance and pain at T2. Hyperarousal at T1 predicted intrusions at T2. Pain at T1 predicted all PTSD symptom clusters at T2, although the association with hyperarousal was only significant within the 90% CI. Intrusions at T2 predicted hyperarousal and pain at T3. And pain at T2 predicted hyperarousal at T3 but was not related to intrusions or avoidance at T3. Cross-sectional associations between pain and acute PTSD symptom clusters were all significant at T1 within the 95% CI but at T2 and T3, associations were only significant within the 90% CI and one association at T2 (intrusions and pain) was not significant. This model produced acceptable fit indices: CFI = 0.979, TLI = 0.949, RMSEA = 0.071, although χ^2^ was still statistically significant (χ^2^ = 51.33, df = 25, *p* = 0.002). Table 2 presents the model estimates and bootstrap bias-corrected confidence intervals.

**Table 2:** Constrained model of pain and PTSD-clusters over time.

		**T2**					**T3**			
		**B**	**SE**	**95% CI**	**β**		**B**	**SE**	**95% CI**	**β**
**Intrusions predicted by**										
Intrusions	T1	**0.38**	**0.11**	**0.17, 0.60**	**0.40**	T2	**0.65**	**0.09**	**0.48, 0.83**	**0.68**
Avoidance	T1					T2				
Hyperarousal	T1	**0.22**	**0.12**	**0.002, 0.46**	**0.22**	T2				
Pain	T1	**0.11**	**0.06**	**0.02, 0.23**	**0.18**	T2				
**Avoidance predicted by**										
Intrusions	T1	**0.22**	**0.11**	**0.01, 0.44**	**0.25**	T2				
Avoidance	T1	**0.26**	**0.09**	**0.08, 0.44**	**0.27**	T2	**0.66**	**0.09**	**0.49, 0.83**	**0.63**
Hyperarousal	T1	0.11	0.11	−0.11, 0.32	0.11	T2				
Pain	T1	**0.10**	**0.05**	**0.01, 0.20**	**0.16**	T2	0.05	0.04	−0.05, 0.13	0.07
**Hyperarousal predicted by**										
Intrusions	T1	0.15	0.12	−0.08, 0.40	0.15	T2	**0.25**	**0.09**	**0.07, 0.43**	**0.0.25**
Avoidance	T1					T2	0.13	0.09	−0.04, 0.32	0.12
Hyperarousal	T1	**0.51**	**0.13**	**0.27, 0.75**	**0.47**	T2	**0.31**	**0.10**	**0.11, 0.49**	**0.33**
Pain	T1	*0.10*	*0.06*	–*0.01, 0.21*	*0.14*	T2	**0.10**	**0.05**	**0.002, 0.18**	**0.13**
**Pain predicted by**										
Intrusions	T1	**0.39**	**0.15**	**0.9, 0.69**	**0.30**	T2	**0.54**	**0.19**	**0.16, 0.87**	**0.41**
Avoidance	T1	−0.26	0.18	−0.62, 0.10	−0.18	T2				
Hyperarousal	T1					T2	−0.26	0.17	−0.58, 0.09	−0.21
Pain	T1	**0.47**	**0.09**	**0.30, 0.64**	**0.53**	T2	**0.49**	**0.10**	**0.33, 0.74**	**0.52**

*T1 = in-hospital, T2 = 3 months post-burn, T3 = 6 months post-burn, CI = Bootstrap bias-corrected two-sided 95% confidence interval. Statistically significant effects within the 90% confidence interval are written in italics. Statistically significant relationships were printed in bold*.

## Discussion

This study investigated the relationship between (acute) PTSD symptom clusters and pain in a sample of patients with burns. As expected, results showed that acute PTSD symptoms predicted later PTSD symptoms. Likewise, the level of acute pain was a predictor of later chronic pain. Beyond these stability paths, our study showed that in the early post-burn phase, pain and PTSD symptom clusters were entangled. In particular, there was a bidirectional relationship between intrusions and pain in the first wave. Unidirectional relations were found for pain to avoidance and, to a lesser extent, hyperarousal. In the second wave, there was an unidirectional relation from intrusions to pain, and from pain to hyperarousal. This partly supports mutual maintenance shortly after the burn event through intrusions, but not after 3 months. The role of intrusions in connection with pain is in line with the Perpetual Avoidance Model but a role of avoidance could not be substantiated.

This study supports an entangled influence of pain and PTSD symptoms but a mutually maintaining relationship between pain and PTSD symptoms over time could not be established. The central role of intrusions in the PTSD-pain connection is in concert with the Perpetual Avoidance Model (Liedl and Knaevelsrud, 2008) which assumes that intrusions following a traumatic event play a crucial role in inducing vicious circles of PTSD symptoms and pain. The finding that intrusions consistently predicted pain in the two waves, as was also reported by Liedl et al. (2010) may subscribe this effect. PTSD driving the presence of pain was earlier reported by Brown et al. (2014) and Jenewein et al. (2009). The finding that particularly intrusions predicted subsequent pain, suggests that high levels of intrusions after a traumatic burn event may be important in the development of chronic pain.

This study also showed a relationship between baseline pain and cross-sectional and subsequent intrusions, avoidance and hyperarousal in the first wave, and an unidirectional relationship between pain and subsequent hyperarousal in the second wave. This suggests that acute pain has the potency to reinforce acute and later PTSD symptoms. In the second wave, an unidirectional relationship between pain and subsequent hyperarousal was found. The significant role of hyperarousal was also reported by Liedl et al. (2010), but the effect was less pronounced in our study. Whereas, Liedl et al. (2010) found a bidirectional relationship between pain and hyperarousal, our study could not replicate this relationship. Differences in timing of measurement and severity of symptom levels may provide an explanation for the lack of unequivocal results.

While an unidirectional relationship was found between baseline pain and subsequent avoidance, this relationship disappeared in the second wave. The association between pain and avoidance is in agreement with the Fear-Avoidance Model (e.g., Vlaeyen and Linton, 2012) and with the Perpetual Avoidance Model proposing that avoidance behavior is involved in the development and maintenance of pain. The finding that this relationship disappeared may be explained by the decrease in pain and PTSD symptoms over time, thereby diluting the strength of the association. Of notice, this study used the PTSD-related avoidance cluster which may tap into another construct compared to pain-related avoidance. Measuring avoidance behavior related to pain may be more powerful to find an effect. Another explanation may be the relatively small sample size of our study and the low levels of chronic pain which may cause a power problem, as the role of avoidance in the maintenance of pain is well-documented. However, also a review study concluded that avoidance was not central in the reciprocity of PTSD and pain (Ravn et al., 2018).

This study has some limitations. First, we measured PTSD symptoms using a self-report questionnaire where a diagnostic interview is preferred when assessing PTSD (Engelhard et al., 2007). However, prior studies have indicated the sensitivity of using self-report questionnaires, including the IES in burn populations (Sveen et al., 2010). Second, three symptom clusters of PTSD (DSM-IV) were assessed, rather than the four symptom clusters as defined in DSM-5. Third, we reported results significant within the 90% CI, which are considered a weaker relationship. Fourth, some model fit indices suggested some misspecification of the saturated model. To improve model fit, paths were constrained to zero based on a data driven approach. Furthermore, the complexity of the model in a relatively small sample size and relatively low variation in pain and PTSD symptoms, warrants replication of the results. Additionally, less pain scores were obtained compared with PTSD symptoms scores which may violate the assumption that missing values should be random, when being imputed. Fifth, we did not assess other meaningful variables that may influence pain and PTSD.

Although the results of this study can only be interpreted as correlative, the study may have relevant implications for further research and treatment. Interventions aimed at reducing intrusions in the early phase after burn injury might have beneficial effects on pain. There is increasing interest in early interventions, including in burn care, which shows promising results (Fauerbach et al., 2020). A wide range of different therapies for PTSD are available (Schnyder et al., 2015) that may be considered. By confronting patients with their traumatic experiences, they learn to reinterpret the situation and label it as part of their past. The “here and now” quality of the reminders may change and the memory more integrated in the autobiographical memory (Liedl and Knaevelsrud, 2008). At the same time, more efforts to further improve pain management in burn care, e.g., by implementing non-pharmacological pain interventions (de Jong et al., 2007) may have a beneficial effect on PTSD symptoms. Further research is needed to investigate these hypotheses and the possible beneficial effect of psychological and pain interventions.

In conclusion, this study showed that in the post-burn phase, pain and PTSD symptom clusters are entangled. A temporal mutual maintenance between pain and intrusions was found, providing some support for Sharp and Harvey's Mutual Maintenance Model and for the role of intrusions in the Perpetual Avoidance Model (Liedl and Knaevelsrud, 2008). The central role of avoidance, however, was not supported, contrasting that part of the Perpetual Avoidance Model and the Fear-Avoidance Model (Vlaeyen and Linton, 2012). In sum, particularly intrusions were driving the presence of pain, which may call attention for interventions focused on intrusions in an attempt to break the vicious pain-PTSD circle.

## Data Availability Statement

The raw data supporting the conclusions of this article will be made available by the authors upon request, without undue reservation.

## Ethics Statement

The studies involving human participants were reviewed and approved by Medisch Ethische Toetsingscommissie Noord-Holland, the Netherlands and Commissie voor Medische ethiek, Universiteit Gent, Belgium. The patients/participants provided their written informed consent to participate in this study.

## Author Contributions

Data were analyzed by NVL. The first draft of the manuscript was written by VdV under supervision of NVL. All authors contributed to the study conception and design, and/or data collection, commented on the manuscript, read, and approved the final manuscript.

## Conflict of Interest

The authors declare that the research was conducted in the absence of any commercial or financial relationships that could be construed as a potential conflict of interest.
