# Supplementary material for: Pain and Posttraumatic Stress Symptom Clusters: A Cross-Lagged Study
Source: Front Psychol. 2021 May 31;12:669231. doi: 10.3389/fpsyg.2021.669231 (PMC8201070; doi:10.3389/fpsyg.2021.669231)
Supplement: Supplementary file 1 [file Data_Sheet_1.docx]

Appendix 1. Estimates of pain and PTSD-clusters in a saturated model

|  |  | T2 |  |  |  |  | T3 | |  |
| --- | --- | --- | --- | --- | --- | --- | --- | --- | --- |
|  |  | B | SE | p-value |  | B | | SE | p-value |
| Intrusions predicted by |  |  |  |  |  |  | |  |  |
| Intrusions | T1 | 0.390 | 0.122 | .001 | T2 | 0.559 | | 0.175 | .001 |
| Avoidance | T1 | -0.023 | 0.113 | .839 | T2 | 0.034 | | 0.145 | .813 |
| Hyperarousal | T1 | 0.227 | 0.123 | .064 | T2 | 0.097 | | 0.129 | .451 |
| Pain | T1 | 0.116 | 0.056 | .037 | T2 | 0.016 | | 0.047 | .733 |
| Avoidance predicted by |  |  |  |  |  |  | |  |  |
| Intrusions | T1 | 0.225 | 0.111 | .043 | T2 | 0.012 | | 0.164 | .941 |
| Avoidance | T1 | 0.261 | 0.107 | .014 | T2 | 0.661 | | 0.177 | .000 |
| Hyperarousal | T1 | 0.102 | 0.113 | .370 | T2 | 0.026 | | 0.141 | .852 |
| Pain | T1 | 0.096 | 0.049 | .052 | T2 | 0.062 | | 0.060 | .302 |
| Hyperarousal predicted by |  |  |  |  |  |  | |  |  |
| Intrusions | T1 | 0.128 | 0.134 | .339 | T2 | 0.206 | | 0.151 | .174 |
| Avoidance | T1 | 0.072 | 0.121 | .550 | T2 | 0.148 | | 0.153 | .333 |
| Hyperarousal | T1 | 0.474 | 0.133 | .000 | T2 | 0.367 | | 0.159 | .021 |
| Pain | T1 | 0.092 | 0.056 | .103 | T2 | 0.107 | | 0.062 | .083 |
| Pain predicted by |  |  |  |  |  |  | |  |  |
| Intrusions | T1 | 0.417 | 0.203 | .040 | T2 | 0.552 | | 0.207 | .008 |
| Avoidance | T1 | -0.213 | 0.196 | .278 | T2 | -0.040 | | 0.177 | .823 |
| Hyperarousal | T1 | -0.079 | 0.206 | .701 | T2 | -0.232 | | 0.193 | .228 |
| Pain | T1 | 0.468 | 0.089 | .000 | T2 | 0.499 | | 0.104 | .000 |

Note. T1 = in-hospital, T2 = 3 months post burn, T3 = 6 months post burn
